# Severe acute respiratory syndrome coronavirus 2 (SARS-CoV-2) nucleic acid contamination of surfaces on a coronavirus disease 2019 (COVID-19) ward and intensive care unit

**DOI:** 10.1017/ice.2020.416

**Published:** 2020-08-12

**Authors:** Sarah N. Redmond, Khalid M. Dousa, Lucas D. Jones, Daniel F. Li, Jennifer L. Cadnum, Maria E. Navas, Nataliya M. Kachaluba, Sandra Y. Silva, Trina F. Zabarsky, Elizabeth C. Eckstein, Gary W. Procop, Curtis J. Donskey

**Affiliations:** 1 Case Western Reserve University School of Medicine, Cleveland, Ohio; 2 Geriatric Research, Education, and Clinical Center, Louis Stokes Cleveland VA Medical Center, Cleveland, Ohio; 3 Department of Molecular Biology and Microbiology, Case Western Reserve University School of Medicine, Cleveland, Ohio; 4 Research Service, Louis Stokes Cleveland VA Medical Center, Cleveland, Ohio; 5 Pathology and Laboratory Medicine Services, Louis Stokes Cleveland VA Medical Center, Cleveland, Ohio; 6 Clinical and Translational Science Program, School of Medicine, Case Western Reserve University, Cleveland, Ohio; 7 Infection Control Department, Louis Stokes Cleveland VA Medical Center, Cleveland, Ohio; 8 Pathology and Laboratory Medicine Institute, Cleveland Clinic Foundation, Cleveland, Ohio

## Abstract

On coronavirus disease 2019 (COVID-19) wards, severe acute respiratory syndrome coronavirus 2 (SARS-CoV-2) nucleic acid was frequently detected on high-touch surfaces, floors, and socks inside patient rooms. Contamination of floors and shoes was common outside patient rooms on the COVID-19 wards but decreased after improvements in floor cleaning and disinfection were implemented.

Transmission of respiratory viruses, including severe acute respiratory syndrome coronavirus 2 (SARS-CoV-2), commonly occurs through exposure to respiratory droplets.^1,2^ The importance of contaminated surfaces in transmission is uncertain.^2^ However, respiratory viruses can survive for hours to days on surfaces.^1,3^ Therefore, minimizing contact with surfaces, hand hygiene after surface contact, and environmental cleaning and disinfection are recommended to reduce risk for acquisition of SARS-CoV-2.^2^

In healthcare facilities, surfaces in rooms of patients with coronavirus disease 2019 (COVID-19) are frequently contaminated with SARS-CoV-2 nucleic acid.^4^ Widespread SARS-CoV-2 nucleic acid contamination outside the rooms of infected patients has also been reported.^5,6^ To develop effective interventions, there is a need for additional data on environmental contamination. Here, we examined SARS-CoV-2 contamination of surfaces on a COVID-19 ward and intensive care unit (ICU) and used the results to implement cleaning and disinfection interventions.

## Methods

### Study setting

The Cleveland VA Medical Center is a 215-bed hospital with a 22-bed COVID-19 ward and 8-bed COVID-19 ICU. The ICU includes 8 beds for care of non–COVID-19 patients, but with separate primary nursing and physician teams. COVID-19 patients are restricted to their rooms unless they must leave for procedures. Dedicated environmental services personnel are assigned to clean COVID-19 rooms on the ward and in the ICU, and policies and procedures are the same for both units.

### Environmental sampling and processing

The study was approved by the Cleveland VA Medical Center Institutional Review Board. We conducted 3 point-prevalence environmental surveys over 5 weeks. Premoistened CLASSIQSwabs with universal transport medium (Copan Diagnostics, Murrieta, CA) were used to sample surfaces in COVID-19 rooms, on the COVID-19 wards outside COVID-19 rooms, and from 2 non–COVID-19 wards. In patient rooms, sites included high-touch surfaces (5 × 20-cm areas of the bed rail and bedside table), floors (5 × 20-cm areas), and socks of COVID-19 patients. Outside patient rooms, sites sampled included portable equipment, nursing stations (eg, telephones, countertops, computers), floors, and shoe soles of personnel. Samples were collected in the late afternoons.

The specimens were tested in the Cleveland Clinic Foundation’s clinical laboratory using the Centers for Disease Control and Prevention’s SARS-CoV-2 reverse transcription polymerase chain reaction (RT-PCR) test.^7^ Only positive test results were included in the analysis. A detailed description of the testing methods is included as Supplementary Material (online).

### Environmental cleaning and disinfection interventions

At the time of the initial point-prevalence survey, a detergent was used for floors, a sodium hypochlorite disinfectant was used daily and after discharge for nonfloor surfaces in patient rooms, and a hydrogen peroxide disinfectant was used by nursing and environmental services staff for nonfloor surfaces outside patient rooms. Daily disinfection of surfaces in patient rooms was performed in the morning. After review of initial results, a quaternary ammonium-based disinfectant was substituted for the detergent for floors; the second point-prevalence survey occurred 2 weeks after the product substitution. After review of the second set of results, a protocol for increased frequency and thoroughness of cleaning and disinfection of floors was implemented, including intermittent use of a disinfectant containing 2,500 ppm sodium hypochlorite. The third set of samples was collected 1 week after the protocol change.

### Data analysis

The Fisher exact test was used to compare proportions. The nonparametric Mann-Kendall test was used to detect trends in the frequency of contamination. All analyses were performed using R version 3.5.1 statistical software (The R Foundation for Statistical Computing, Vienna, Austria).

## Results

**Fig. 1.**
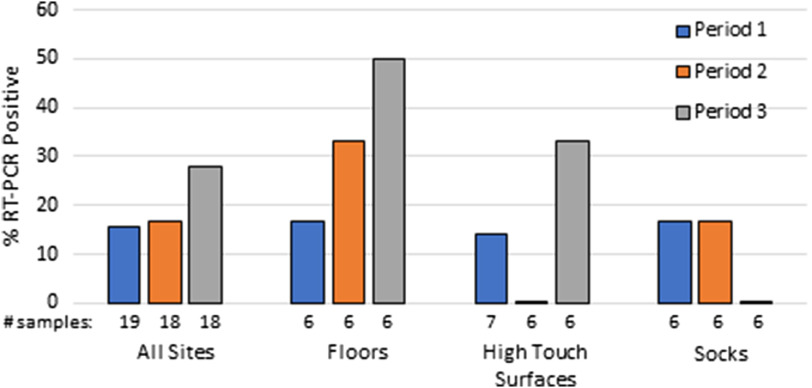
Point prevalence of severe acute respiratory virus coronavirus 2 (SARS-CoV-2) nucleic acid detection on surfaces inside coronavirus 2019 (COVID-19) patient rooms during 3 periods with differing cleaning protocols described in the text. Note. RT-PCR, reverse transcription polymerase chain reaction.

In total, 150 sites were sampled: 55 samples inside COVID-19 rooms, 71 samples on the COVID-19 units outside COVID-19 rooms, and 24 samples from areas outside the COVID-19 ward. The frequency of contamination at all sites inside COVID-19 patient rooms did not differ for the 3 periods (*P* > .05) (Fig. 1). Overall, contamination was more common on floors than on high-touch surfaces, but the difference was not statistically significant: 6 of 18 (33.3%) versus 3 of 19 (15.8%) (*P* = .27). Two rooms sampled after postdischarge cleaning had negative results.

**Fig. 2.**
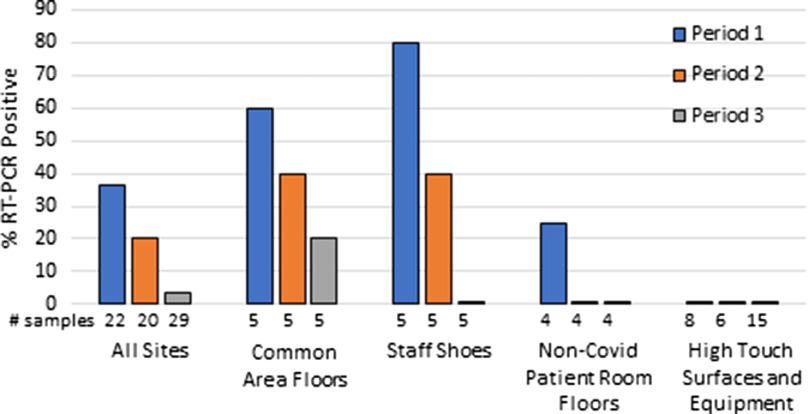
Point prevalence of severe acute respiratory virus coronavirus 2 (SARS-CoV-2) nucleic acid detection on surfaces outside coronavirus 2019 (COVID-19) patient rooms during 3 periods with differing cleaning protocols described in the text. Note. RT-PCR, reverse transcription polymerase chain reaction.

Figure 2 shows the frequency of contamination on the COVID-19 wards outside of COVID-19 rooms. Trend analysis demonstrated a significant reduction in contamination of all sites from period 1 through period 3 from 36.4% to 20.0% to 3.4% (*P* = .007). One non–COVID-19 room in the ICU had floor contamination during the initial survey. All 26 samples from high-touch surfaces and portable equipment were negative.

All 24 samples collected outside the COVID-19 units were negative, including 3 nursing stations and 21 floor samples including in the hallways exiting the COVID-19 units, non-COVID-19 ward hallways, and elevators.

Cycle threshold values were available for the first sampling period. The mean cycle thresholds for the N1, N2, and N3 targets were 30.6 (range, 28.1–33.6), 31.1 (range, 28.7–34.7), and 30.1 (range, 29.6–30.9), respectively.

## Discussion

Our results are consistent with previous reports of frequent SARS-CoV-2 nucleic acid contamination in rooms of COVID-19 patients.^4^ In contrast to some recent reports,^5,6^ we did not detect contamination on high-touch surfaces or portable equipment outside COVID-19 patient rooms. However, SARS-CoV-2 contamination was common on floors and shoes at the time of the initial survey. Interventions to improve floor cleaning and disinfection reduced contamination of floors and shoes outside COVID-19 rooms.

The absence of SARS-CoV-2 nucleic acid on high-touch surfaces and equipment outside of COVID-19 rooms suggests that intensive cleaning protocols and use of PPE can minimize the risk for transfer of SARS-CoV-2 from patient rooms to these surfaces. COVID-19 patients were in contact precautions. Nursing and environmental services personnel performed frequent disinfection of high-touch surfaces and equipment.

The frequent floor contamination at the time of the initial point-prevalence survey may be attributable to infrequent cleaning and use of a detergent rather than a disinfectant. In that setting, shoes of personnel may serve as a vector for transfer of SARS-CoV-2 from floors in COVID-19 rooms to floors throughout the unit. The importance of floor contamination in SARS-CoV-2 transmission is uncertain. However, in a simulation study, a benign bacteriophage inoculated on floors in patient rooms disseminated widely to surfaces inside and outside the rooms.^8^ Also, high-touch objects in hospitals often contact floors and can serve as a source for hand contamination.^9^ Based on these findings, it is reasonable to consider measures to minimize dissemination of SARS-CoV-2 from floors.

Our study has some limitations. The PCR assay does not distinguish between viable and nonviable viral particles. In a laboratory study, viable influenza viruses inoculated onto surfaces were recovered for up to 2 weeks, but RNA was detected by PCR for 7 weeks.^10^ Recent studies have not recovered viable SARS-CoV-2 from environmental sites with positive PCR results.^6^ The study was conducted in 1 hospital, and given the small number of COVID-19 rooms sampled, we were unable to assess the impact of factors such as viral load, patient mobility and symptoms, and level of medical care on contamination. Larger studies are therefore needed in other healthcare facilities. The interventions did not result in reduced contamination of floors inside COVID-19 rooms, possibly due to ongoing shedding of virus particles. Finally, we did not evaluate alternative approaches to reduce dissemination from floors such as use of shoe covers inside COVID-19 rooms.

In conclusion, SARS-CoV-2 nucleic acid was frequently detected on floors and high-touch surfaces inside COVID-19 rooms and on floors and shoes outside patient rooms on COVID-19 units. Simple modifications of floor cleaning and disinfection protocols could be effective in reducing floor and shoe contamination.

## Supplementary material

For supplementary material accompanying this paper visit http://dx.doi.org/10.1017/ice.2020.416. click here to view supplementary material
