# Supplementary material for: Severe acute respiratory syndrome coronavirus 2 (SARS-CoV-2) nucleic acid contamination of surfaces on a coronavirus disease 2019 (COVID-19) ward and intensive care unit
Source: Infect Control Hosp Epidemiol. 2020 Aug 12:1–3. doi: 10.1017/ice.2020.416 (PMC7511842; doi:10.1017/ice.2020.416)
Supplement: Supplementary file 1 [file S0899823X2000416Xsup001.docx]

**Supplemental file.**

**Reverse transcription polymerase chain reaction (RT-PCR) protocol for detection of SARS-CoV-2 nucleic acid**

The specimens were predominantly tested using the original Centers for Disease Control and Prevention SARS-CoV-2 reverse transcription polymerase chain reaction (RT-PCR) test.^1^ This test consists of four individual RT-PCR assays, three of which target the different loci within the nucleocapsid (N) gene and one of which targets a portion of the human RNase P gene. The latter assay is included as an amplification control for human specimens, and relevant to this study demonstrates the presence of human biomass. 200 µl of specimen-containing viral transport media was inactivated with 200 µl of MagNA Pure Bacterial Lysis Buffer (Roche Diagnostics, Indianapolois, IN). The nucleic acids from 200 µl of the inactivated specimen were extracted using the MagNA Pure system (Roche). Five microliters of the eluate was added to 15 µl of mastermix and the RT-PCR reaction was performed in the ABI 7500 or ABI 7500 Fast Dx (Thermofisher, Waltham, MA). All three N gene RT-PCR reactions were required to be positive for the specimen to be reported as positive. All three N gene RT-PCR reactions were required to be negative in the presence of amplification of the human RNase P gene for the specimen to be reported as specimen. Specimens with < 3 N gene amplification products were reported as Indeterminate and those with no RNase P gene amplification were Invalid. Specimens with Indeterminate or Invalid reactions were submitted for resolution testing using the Simplexa® COVID-19 Direct Kit (DiaSorin, Stillwater, MN).

The Simplexa® COVID-19 Direct Kit (DiaSorin) assay was performed according to the manufacturer's instructions. Fifty microliters of Simplexa COVID-19 Direct Kit reaction mix and 50 µl of non-extracted environmental swab sample were used. Data collection and analysis was performed with LIAISON® MDX Studio software (DiaSorin). The assay targets the Surface (S) gene and Open Reading Frame 1ab (ORF1ab) with an RNA internal control (Q670 probe) is used to resolve RT-PCR failure and/or inhibition. The results interpretation algorithm for reporting a positive specimen requires only one of the two targets to be detected within fewer than 40 cycles.

**Reference**

1. Centers for Disease Control and Prevention Division of Viral Diseases. 2019-Novel Coronavirus (2019-nCoV) Real-Time RT-PCR Diagnostic Panel. https://www.fda.gov/media/134922/downloadCDC PRC protocol. Accessed 6/1/2020.
